# Numerical study on the three-dimensional temperature distribution according to laser conditions in photothermal therapy of peri-implantitis

**DOI:** 10.1186/s40729-024-00537-y

**Published:** 2024-04-24

**Authors:** Jeeyong Paik, Donghyuk Kim, Hyunjung Kim, Hee-Sun Kim

**Affiliations:** 1 Department of Mechanical Engineering, Ajou University, 16499 Suwon-si, Gyeonggi-do Korea; 2 Department of Dentistry, SMG_SNU Boramae Medical Center, 07061 Seoul, Korea

**Keywords:** Dental implants, Peri-implantitis, Photothermal therapy, Inflammation, Numerical analysis, Computer-assisted, Computer simulation

## Abstract

**Purpose:**

Dental implants have been successfully implemented as a treatment for tooth loss. However, peri-implantitis, an inflammatory reaction owing to microbial deposition around the implant, can lead to implant failure. So, it is necessary to treat peri-implantitis. Therefore, this numerical study is aimed at investigating conditions for treating peri-implantitis.

**Methods:**

Photothermal therapy, a laser treatment method, utilizes photothermal effect, in which light is converted to heat. This technique has advantage of selectively curing inflamed tissues by increasing their temperature. Accordingly, herein, photothermal effect on peri-implantitis is studied through numerical analysis with using Arrhenius damage integral and Arrhenius thermal damage ratio.

**Results:**

Through numerical analysis on peri-implantitis treatment, we explored temperature changes under varied laser settings (laser power, radius, irradiation time). We obtained the temperature distribution on interface of artificial tooth root and inflammation and determined whether temperature exceeds or does not exceed 47℃ to know which laser power affects alveolar bone indirectly. We defined the Arrhenius thermal damage ratio as a variable and determined that the maximum laser power that does not exceed 47℃ at the AA’ line is 1.0 W. Additionally, we found that the value of the Arrhenius thermal damage ratio is 0.26 for a laser irradiation time of 100 s and 0.50 for 500 s.

**Conclusion:**

The result of this numerical study indicates that the Arrhenius thermal damage ratio can be used as a standard for determining the treatment conditions to help assisted laser treatment for peri-implantitis in each numerical analysis scenario.

## Introduction

In patients with tooth loss, despite the extensive and demanding procedures as well as the considerable time and expense involved, the successful completion of implant placement surgery and the installation of the superstructure necessitates ongoing maintenance for the longevity and healthy use of the implant [1]. Peri-implantitis is an inflammation involving bone loss around the implant tissue owing to microbial infection, analogous to periodontitis in natural teeth [2–6]. Initially, the accumulation of biofilm on the implant surface leads to biofilm proliferation. This results in the formation of plaques on the implant surface, and as the gap between the gum and the implant starts to widen, inflammation occurs in the surrounding gum tissue. If this issue is unresolved, the biofilm can cause an inflammatory response that extends from the surrounding tissue to the inner bone, leading to the progressive destruction of the bone around the implant [7–10]. Peri-implantitis is known as the major cause of late-stage implant failure; while the treatment of the biofilm is essential for treatment, an ideal method for sterilizing the implant surface and restoring the health of the surrounding tissue has not yet been established [11–14].

Various methods, such as physical treatment using plastic curettes, local or systemic administration of antibiotics, local antiseptic treatments using chlorhexidine, and the use of air-abrasive devices, have been employed to treat the biofilm around implants [15–18]. However, mechanical treatment alone often fails to eliminate the biofilm [14, 19], and the use of chemical agents or antibiotics can be minimally effective or raise concerns regarding side effects [20]. Therefore, recent studies have focused on the use of dental lasers to sterilize and purify implant surfaces [21–23]. The laser could be used in either secondary prevention or raising levels of prevention for peri-implantitis [24]. This approach has advantages over traditional methods, including a lower likelihood of bleeding, faster recovery, and greater penetration depth, which facilitate the treatment of inflammation deep within the gingiva.

Photothermal therapy (PTT) is a laser-based method that treats inflammation by increasing the temperature through the heat generated by the laser. PTT operates based on the principle of the photothermal effect [25, 26]. The photothermal effect refers to the phenomenon in which light energy is absorbed by a specific substance and converted into heat [27]. When PTT is used to treat inflammation, the pattern of inflammatory destruction is determined by the location of the inflammation [28]. When inflammation is in direct contact with body tissues, it is necessary to differentiate between apoptosis and necrosis. However, if the inflammation is not in contact with body tissues, the focus can be solely on destruction within the inflammation itself, determining whether the damage is reversible or irreversible. However, if the temperature of the implant surface excessively increases, the heat transferred through the implant can cause thermal damage to the alveolar bone; thus, the temperature of the alveolar bone must be maintained below 47 ℃ [29, 30]. Irreversible tissue damage can be quantitatively assessed using the Arrhenius damage integral [31, 32].

Various studies have been conducted on the changes in the implant surface temperature when a laser is employed [33–35]. Matthias et al. [33] used a continuous-wave 809-nm diode laser to measure the temperature around the implant. The laser was positioned 0.5 mm from the implant surface and irradiated laser power at ranging from 0.5 to 2.5 W in increments of 0.5 W. After irradiation, the time required for the temperature to rise more than 10 ℃ from the initial temperature was measured Alessandro et al. [34] applied 810-nm and 980-nm diode lasers in both, pulsed- and continuous-wave modes to measure temperature changes. In this experiment, the laser was positioned at the upper part of the implant and irradiated (3-mm distance) in laser power 2.0 W and measured temperature changes after an irradiation period of 60 s were compared with before the laser irradiation. Deppe et al. [35] used a 445-nm diode laser to measure the temperature changes of five different types of implants and six irradiation conditions were used. After irradiation, the temperature effects of the 445-nm laser on five different types of implants were assessed. The results showed that, under the same laser intensity and irradiation conditions, the temperature changes varied depending on the type of implant. Although all these studies experimentally measured the temperature of the implant surface, they did not measure the temperature of the inflamed areas of the implant.

Most studies have focused solely on measuring the changes in the implant surface temperature, with insufficient research on the temperature of the inflammation itself [21, 36]. Therefore, in this numerical study, we investigated the effectiveness of various laser irradiation techniques in the adjunctive treatment of peri-implantitis, specifically focusing on identifying laser intensities that ensure the temperature of the surrounding alveolar bone does not exceed 47 ℃. Following this, we employed the Pennes bioheat equation, to accurately calculate the temperature distribution within a cylindrical inflammation area around the implant. Also, using the Arrhenius damage integral, we quantitatively assessed the extent of irreversible inflammation damage and evaluated Arrhenius thermal damage ratio (${\phi }_{Arrh}$) values to find an appropriate value of laser power, radius and irradiation time. Our goal was to ensure an effective approach to peri-implantitis adjunctive treatment by photothermal therapy, while seeking scenarios where the ${\phi }_{Arrh}$ values were maximized without causing harm to the alveolar bone.

## Methods

### Heat transfer model

In this numerical study, after laser irradiation, the Pennes bioheat equation was used to determine the temperature of the surrounding tissues and inflammation around the implant [37]. This equation assumes that the heat generated by blood circulation and metabolic processes is uniformly distributed within the biological tissues. This equation is expressed as Eq. (1): Here, $$ k,\rho,{\text{a}\text{n}\text{d} c}_{p}$$ represent the thermal conductivity, density, and specific heat, respectively. The term$$ {q}_{b}$$ denotes the heat transfer by the blood, whereas $$ {q}_{m}$$ represents the metabolic heat source. $$ {q}_{b}$$ is expressed in Eq. (2), and is calculated based on each material’s blood perfusion rate ($$ {\omega }_{b}$$), blood density ($$ {\rho }_{b}$$), blood specific heat ($$ {c}_{p,b}$$), and the temperature difference between the initial blood temperature ($$ {T}_{b}$$) and the current temperature. Moreover, as there is no blood flow or metabolic activity in the implant, the values of $$ {q}_{b}$$ and $$ {q}_{m}$$ in Eq. (1) become zero, reducing it to a simple heat-diffusion Eq. 

1 $$ \rho {c}_{p}\frac{\partial T}{\partial t}=k{\nabla }^{2}T+{q}_{b}+{q}_{m} $$

2 $$ {q}_{b}={\rho }_{b}{\omega }_{b}{c}_{p,b}({T}_{b}-T)$$

In this numerical study, as heat is applied to inflamed tissues through a 630 nm diode laser, the Pennes bioheat equation in Eq. (1) can be expressed in the form of Eq. (3), which includes a heat source term, $$ {q}_{l}$$, owing to the laser. $$ {q}_{l}$$ is expressed in Eq. (4).

3 $$ \rho {c}_{p}\frac{\partial T}{\partial t}=k{\nabla }^{2}T+{q}_{b}+{q}_{m}+{q}_{l} $$

4 $$ {q}_{l}= {\mu }_{a}\frac{{P}_{l}}{\pi {r}_{l}^{2}}{e}^{-{\mu }_{tot}z}\cdot {e}^{-\frac{{x}^{2}+{y}^{2}}{{r}_{l}^{2}}} ({\mu }_{tot}={\mu }_{a}+{\mu }_{s}^{{\prime }}) $$

Equation (4) is suitable for use when the direction of the laser irradiation aligns with the *z*-direction. However, if the direction of the laser irradiation is inclined, Eq. (4) must be transformed via rotation to account for the angle. The treatment of peri-implantitis involves laser irradiation between the implant and the gingiva, indicating that an inclined laser is more realistic. Therefore, in this numerical study, to formulate the situation of an inclined laser, the positions of *x* and *z* were interchanged, and the concept of rotational transformation, along with differential lengths *dx* and *dz*, was used to consider the angle and position. Additionally, considering the change in the irradiated area owing to the laser’s reflectivity and angle, Eq. (5) was derived in the form presented in [38].

5 $$\begin{aligned} {q}_{l} &= {(1-{R}_{t})\cdot \mu }_{a}\frac{{P}_{l}\cdot cos\theta }{\pi {r}_{l}^{2}}{e}^{-{\mu }_{tot}(-\left(x+dx\right)cos\theta -(z-dz\left)sin\theta \right)} \\ & \quad \cdot {e}^{-\frac{{(-\left(x+dx\right)sin\theta +(z-dz\left)cos\theta \right)}^{2}+{y}^{2}}{{r}_{l}^{2}}} ({\mu }_{tot}={\mu }_{a}+{\mu }_{s}^{{\prime }}) \end{aligned}$$

6 $$ {R}_{t}={R}_{1}+{R}_{2}, {R}_{1}={\left[\frac{\sqrt{{n}_{2}^{2}-{\left({n}_{1}sin\theta \right)}^{2}}-{n}_{1}cos\theta }{\sqrt{{n}_{2}^{2}-{\left({n}_{1}sin\theta \right)}^{2}}+{n}_{1}cos\theta }\right]}^{2} $$

Here, $$ {q}_{l}$$ represents the amount of heat applied to peri-implantitis by the laser, and $$ {R}_{t}$$, $$ {\mu }_{a}$$, $$ {P}_{l}$$, $$ {r}_{l}$$, and $$ \theta $$ correspond to the reflectivity, light absorption coefficient, laser power, laser radius, and irradiation angle, respectively. The attenuation coefficient $$ {\mu }_{tot}$$ is sum of $$ {\mu }_{a}$$ and the reduced scattering coefficient $$ {\mu }_{s}^{{\prime }}$$. The value of $$ {R}_{t}$$ is the sum of the specular reflection value $$ {R}_{1}$$ and the diffuse reflection value $$ {R}_{2}$$. The value for specular reflection is expressed using Eq. (6), and the diffuse reflection value for inflammation at 630 nm was measured as 0.28 [39]. $$ {n}_{1}$$ and $$ {n}_{2}$$ represent the refractive indices of air and inflammation, respectively [40, 41]. Ultimately, by substituting Eq. (5) in Eq. (3), the temperature distribution within the medium (peri-implantitis and implant) can be calculated.

### Arrhenius damage integral & variable

In this numerical study, the final goal of PTT was to maximize the irreversible damage caused by inflammation. Accordingly, the Arrhenius damage integral was utilized to evaluate the extent of damage [28]. This is expressed as presented in Eq. (7). Here, $$ {\Omega }$$ represents the extent of damage to the inflammation, $$ A$$ is the frequency factor, which is a probabilistic variable indicating the likelihood of a reaction, $$ {E}_{a}$$ is the activation energy required to initiate the reaction, $$ R$$ is the ideal gas constant, and $$ T\left(t\right)$$ represents the temperature of the inflammation at time *t* [42, 43].

7 $$ {\Omega }\left(t\right)={\int }_{0}^{t}A{e}^{-\frac{{E}_{a}}{RT\left(t\right)}} dt $$

The Arrhenius damage integral is calculated using the temperature values (K) at each time point, the frequency factor of inflammation $A=2.84\times {10}^{99} {s}^{-1}$, the activation energy of inflammation ${E}_{a}=6.19\times {10}^{5} J/mol$, and the ideal gas constant 8.314 $J/mol \cdot K$, after which the value of ${\Omega }$ is obtained [44, 45]. A value of ${\Omega }$ greater than 1 is considered to indicate irreversible damage. In numerical analysis, the ${\Omega }$ value can be obtained for each grid in all calculated areas, allowing for a quantitative extraction of the extent of irreversible damage within the peri-implantitis. To this end, the volume ratio of the part that has been irreversibly cured compared with the total inflammation is denoted as ${\phi}_{Arrh}$, and this is defined as the Arrhenius thermal damage ratio.

8 $${\phi }_{Arrh}=\frac{Inflammation\,\,Volume\,\,at\,\,{\Omega }\left(t\right)>1}{Total\,\,Inflammation\,\,Volume} $$

### Numerical geometry and properties

**Fig. 1:**
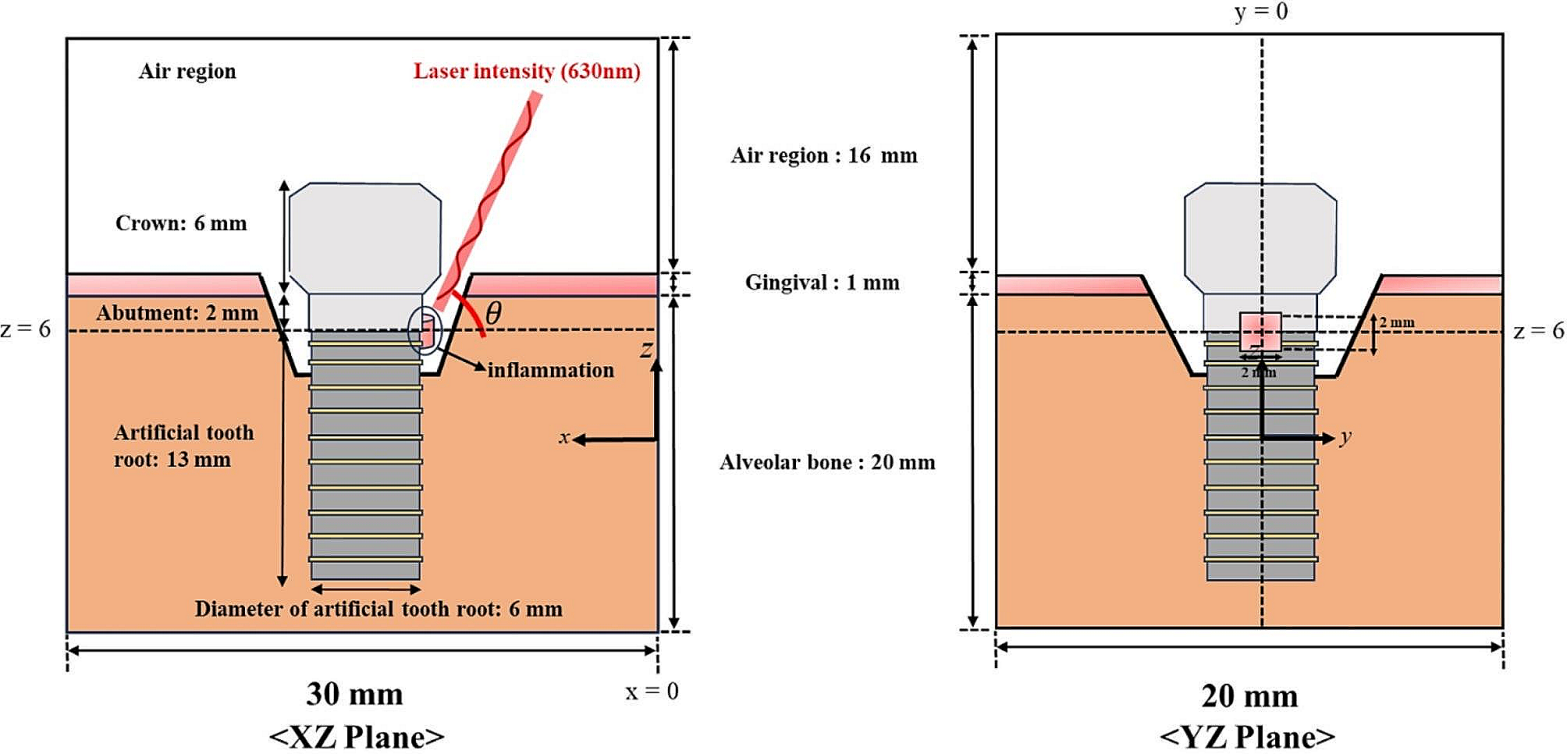
Schematic of the numerical model

**Fig. 2:**
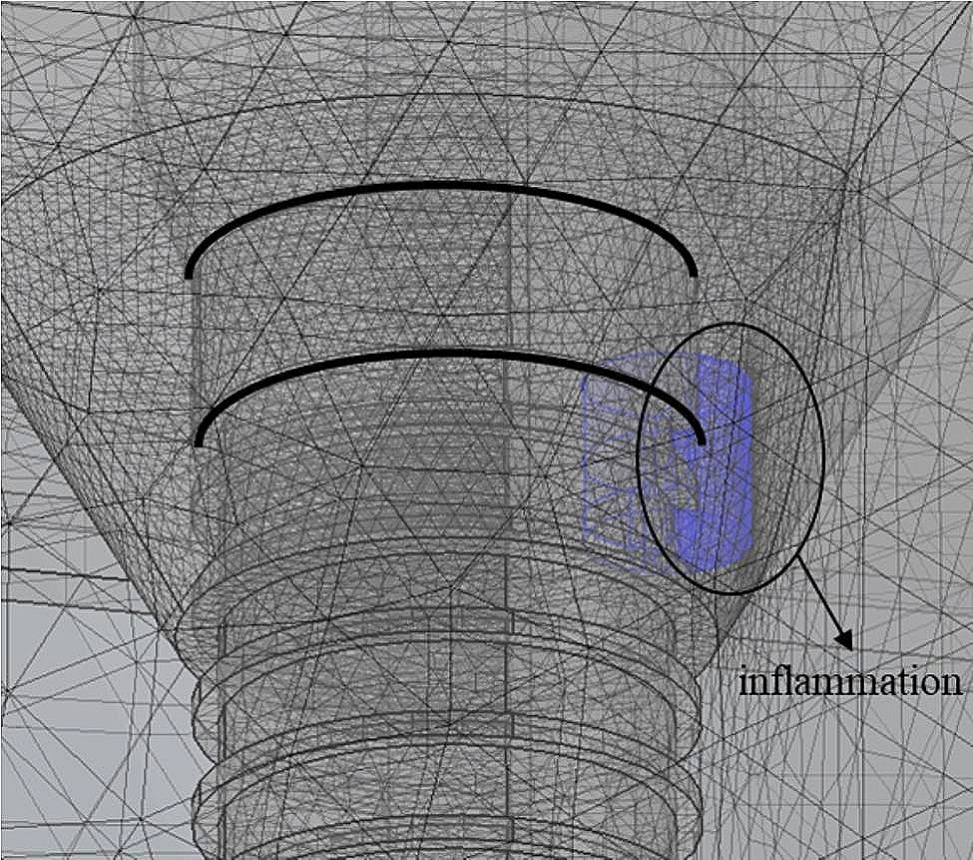
3D model with the grid of the numerical model

In this numerical study, PTT for inflammation caused by implants was evaluated through a numerical analysis. Figure 1 presents a schematic of the numerical model, with the image on the left showing the shape of the 3D model when viewed from the XZ plane and the image on the right showing the same from the YZ plane. Figure 2 illustrates the 3D model with the grid formed in the numerical analysis model. The entire calculation area was set as a rectangular prism encompassing air, gum, implant, and peri-implantitis, with a total height of 37 mm, a width of 30 mm, and a depth of 20 mm; the peri-implantitis was modeled as a cylindrical-shaped inflammation with a height of 2 mm and a diameter of 2 mm, attached to the artificial tooth root and abutment part, as shown in Fig. 2. The model consisted of the crown, gingiva, inflammation, artificial tooth root, abutment connecting the artificial tooth root and crown, alveolar bone, and air region. The wavelength of the irradiated laser was set to 630 nm, and the initial temperature ($$ {T}_{b}$$) of the medium was set at 37 ℃. The thermal and optical properties of each material are summarized in Table 1 [46].

**Table 1:** Various properties of the implant and surroundings [28, 47–56]

	*ρ* (kg/m^3^)	*c* (J/kgK)	*k* (W/mK)	*µa* (1/cm)	*µ’* (1/cm)	*ω*_*b*_ (1/s)	*q*_*m*_ (W/m^3^)
Crown (Zirconia)	6080	450	2.80	0.10	20.43	-	-
Gingival	1000	4200	0.63	0.530	3.817	0.0076	1091
Inflammation	1080	3500	0.48	2.16	17.03	0.009	65,400
Artificial tooth root (Ti-6Al-4 V)	4420	546	7.00	789,500	$\approx 0$	-	-
Abutment (Zirconia)	6080	450	2.80	0.10	20.43	-	-
Alveolar Bone	2060	1260	0.38	0.596	22.97	0.00369	-
Air	1.205	1.006	0.0256	0	0	-	-
Blood	1000	4200	-	-	-	-	-

Table 2 summarizes the numerical conditions. To depict PTT under various conditions, a numerical analysis was conducted across 765 cases, with the laser power ($$ {P}_{l}$$) ranging from 0 to 2.0 W in increments of 0.04 W, the laser radius ($$ {r}_{l}$$) ranging from 0.1 to 0.2 mm in increments of 0.05 mm, and the laser irradiation time (t) ranging from 100 to 500 s in intervals of 100 s. Additionally, the angle of the laser ($$ \theta $$) was fixed at 60°, and the laser was set to operate in the continuous-wave mode.

**Table 2:** Parameters of the numerical model

Parameter	Case	Number	Remarks
Laser power ($$ {P}_{l}$$)	0 to 2.0 W	51	Intv. 0.04 W
Laser radius ($$ {r}_{l}$$)	0.1 to 0.2 mm	3	Intv. 0.05 mm
Laser irradiation time (*t*)	100 to 500 s	5	Intv. 100 s

## Results

### Numerical analysis validation

**Fig. 3:**
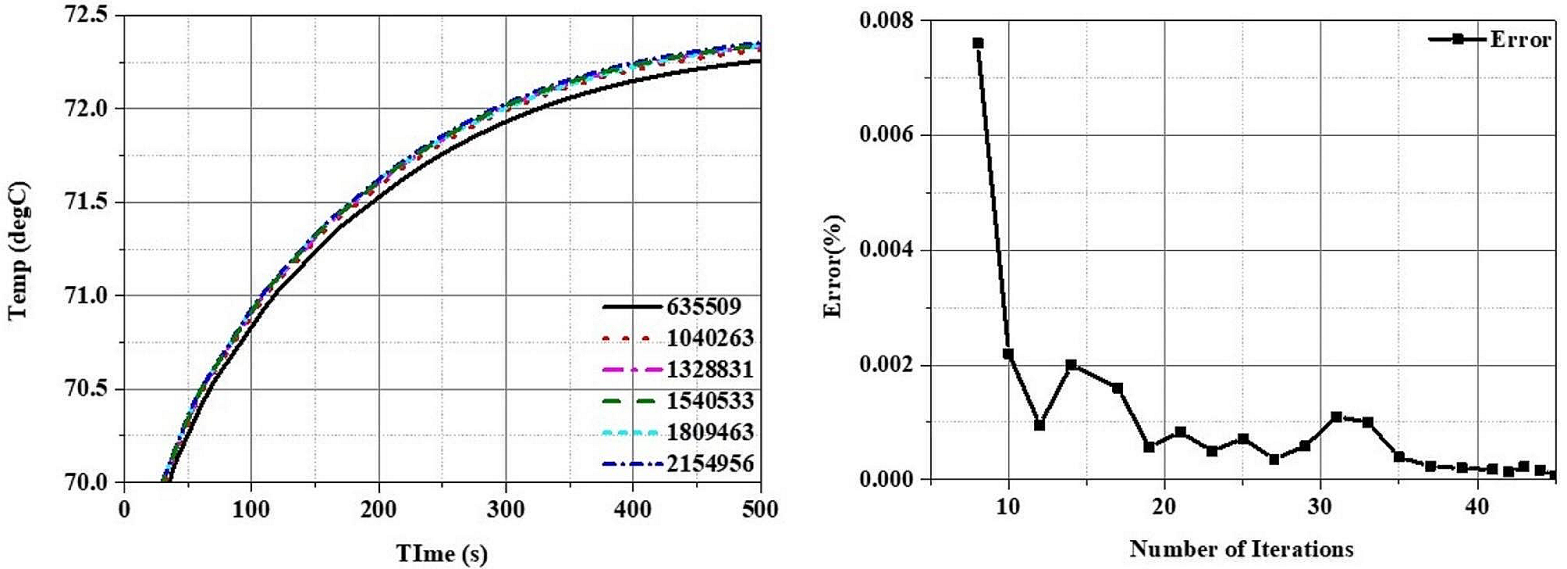
Mesh validation of the numerical model and iteration error in percentage

The analysis tool used in this numerical study was COMSOL Multiphysics, a powerful simulation software based on the finite element method. The backward differentiation formula method was applied to discretize the Pennes Bioheat equation, enabling the calculation of transient temperature distributions over time. Additionally, to validate the numerical analysis model propose herein, a grid independence test and convergence test based on the number of iterations were performed under the laser power of 1.0 W, the laser radius of 0.2 mm, and the irradiation time of 500 s, focusing on the temperature results in the area where the laser first contacts the inflammation. As observed in Fig. 3, when the number of grids reached 1,328,831 or more, the temperature change converged to less than ${10}^{-3}$℃, setting the final number of grids at 1,328,831. Moreover, the error value in relation to the number of iterations dropped below ${10}^{-3}\%$ after 35 iterations, confirming convergence and validating the effectiveness of the numerical analysis model proposed in this numerical study.

### Laser irradiation time and power behavior analysis in inflammation

**Fig. 4:**
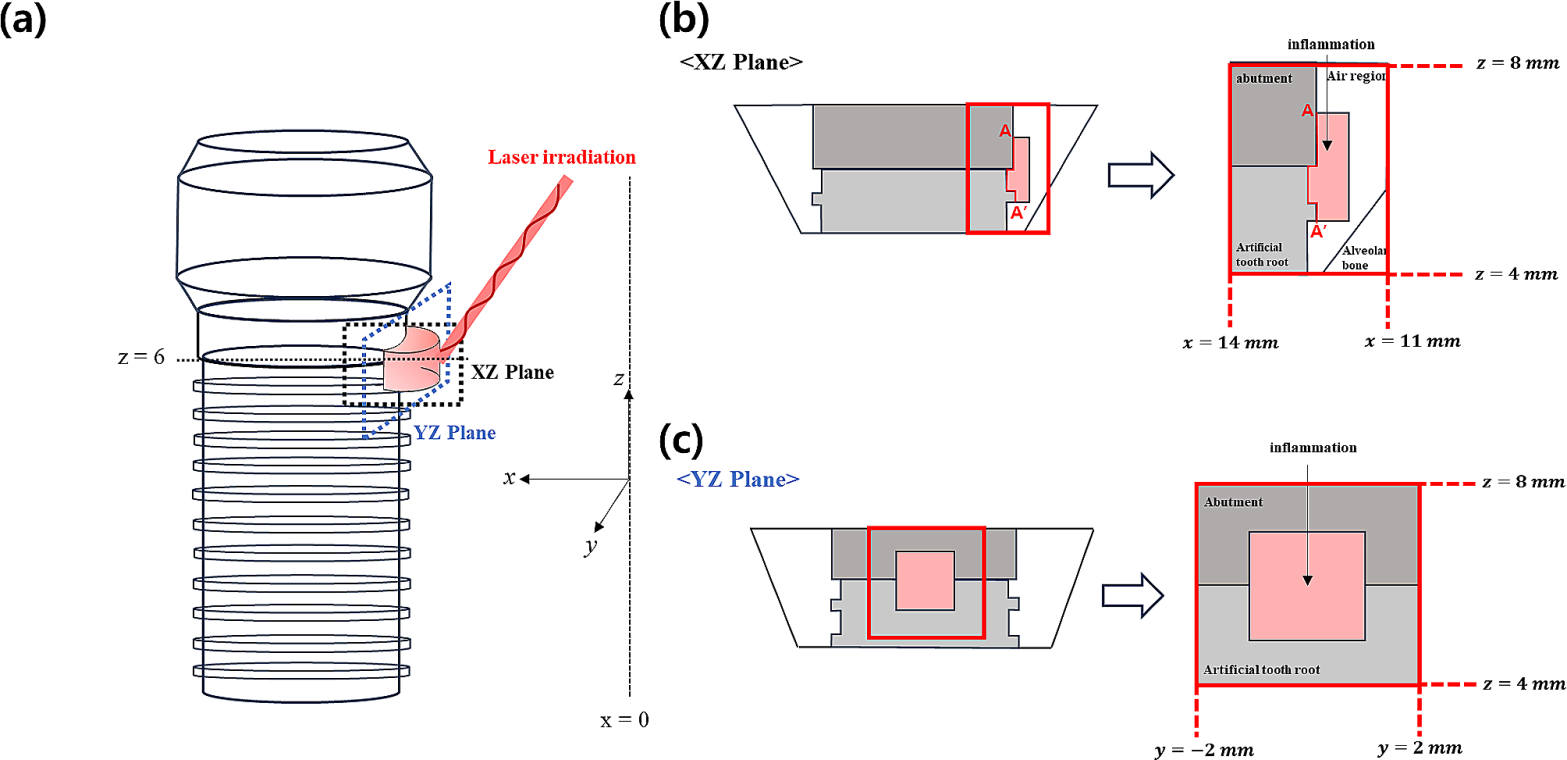
Implant model in various directions

In this numerical study, laser irradiation was focused on the central part of the inflammation (x = 12.3 mm, y = 0 mm, z = 6 mm) to observe the effects of PTT depending on the laser intensity, radius, and irradiation time. To reiterate, the essence of PTT is to increase the temperature of inflammation to maximize irreversible damage. Although the temperature field was calculated in the numerical analysis for the entire model, to focus on the temperature rise in the inflammation owing to the laser, a plot was drawn for the area of x = 11 mm to x = 14 mm, y = -2 mm to y = 2 mm, z = 4 mm to z = 8 mm, as shown in Fig. 4. Figure 4a shows a 3D schematic of the numerical analysis model showing the laser irradiation of the implant and inflamed parts. Figure 4b and c are schematics of the cross-sections in the XZ and YZ directions, respectively, at the inflammation center point (x = 12.8 mm, y = 0, z = 6 mm). In Fig. 4b, the line AA’ represents the tangent to the implant surface where the inflammation, abutment, and artificial tooth root meet.

**Fig. 5:**
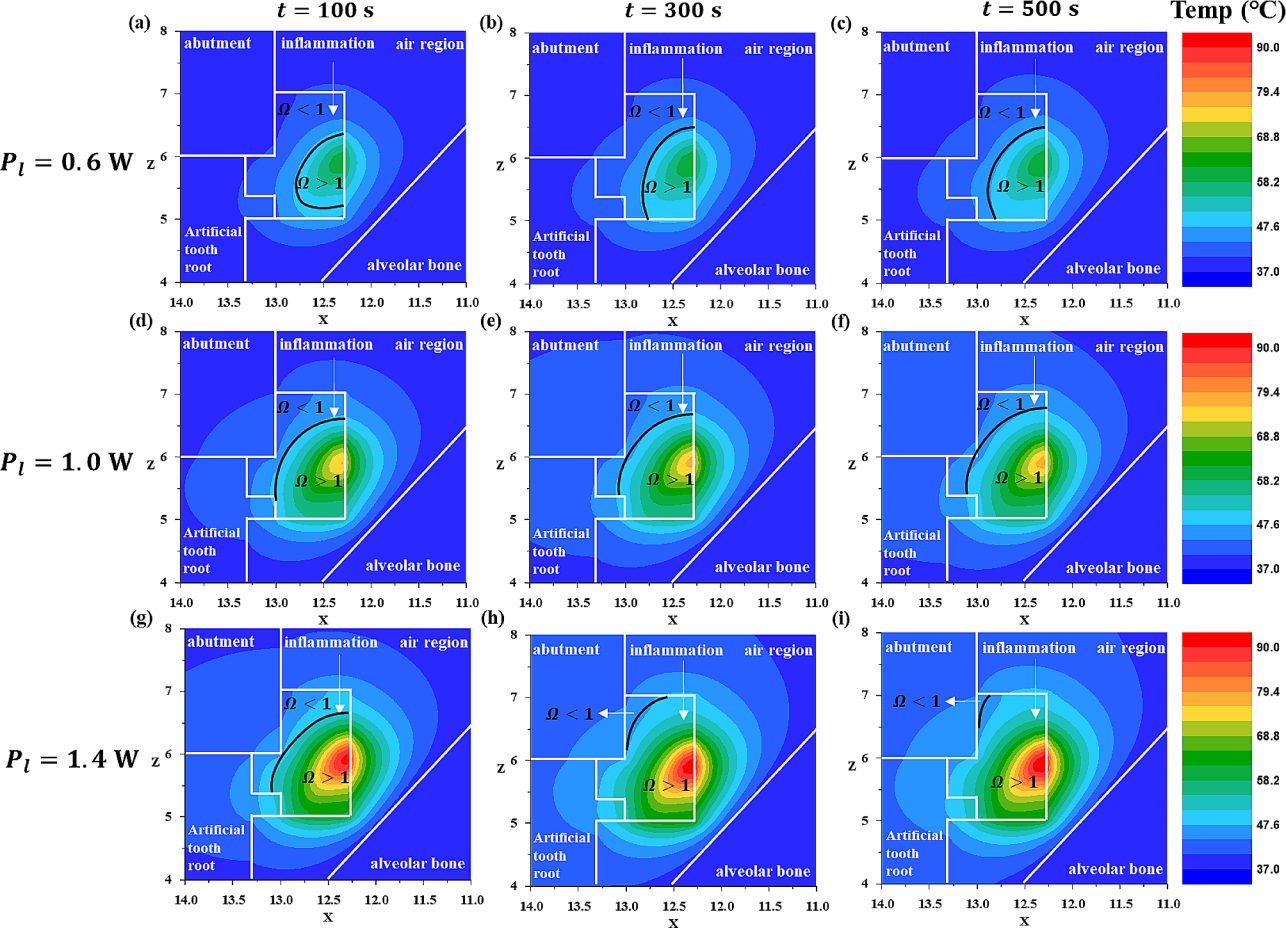
Temperature and ${\Omega }$ distribution with laser irradiation at various laser powers and laser irradiation times (XZ plane direction) for a laer radius of 0.15 mm

**Fig. 6:**
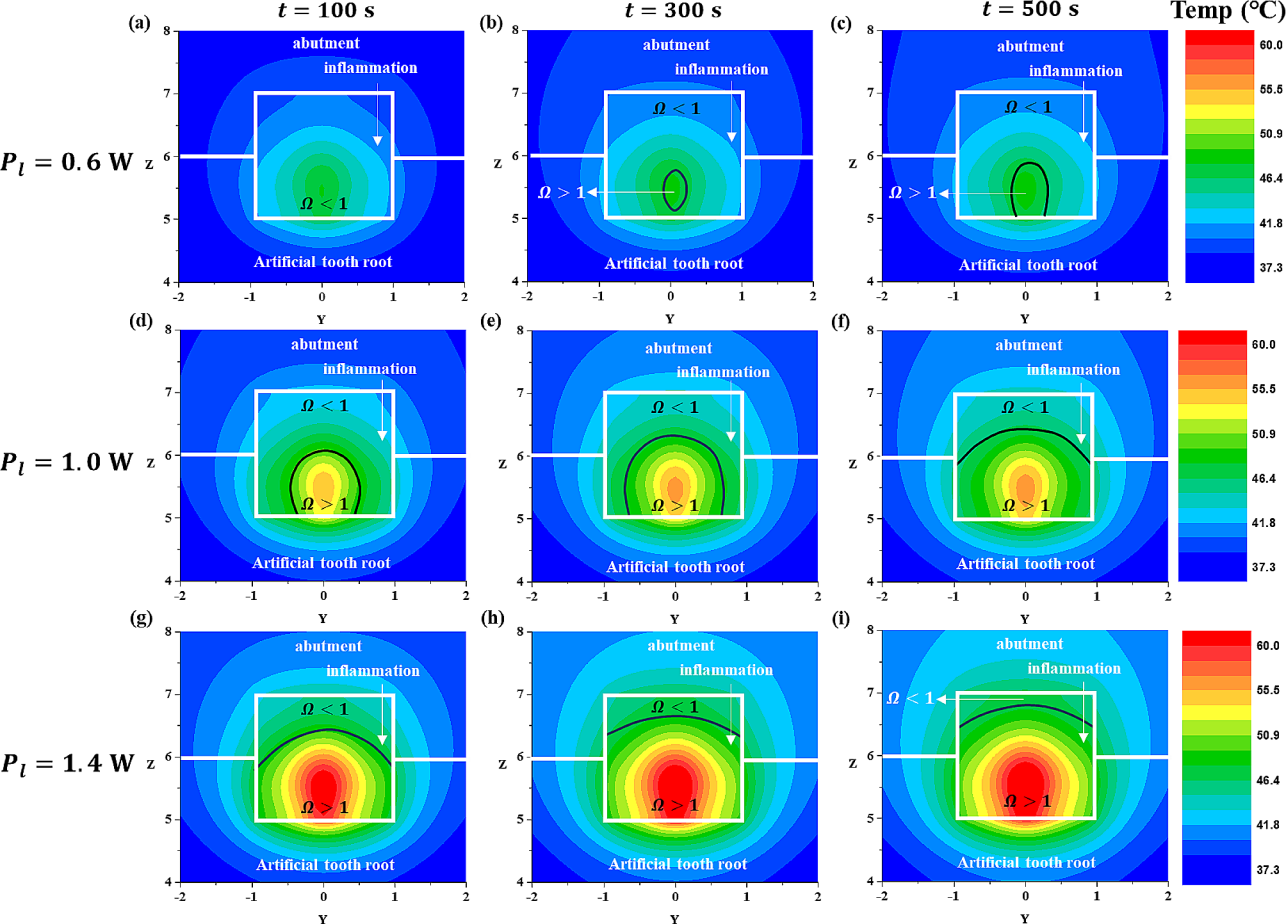
Temperature and ${\Omega }$ distribution with laser irradiation at various laser powers and laser irradiation times for a laser radius of 0.15 mm (YZ plane direction)

First, with the laser radius fixed at 0.15 mm, the laser intensity was set to 0.6 W, 1.0 W, and 1.4 W, and the irradiation time was set to 100 s, 300 s, and 500 s. The temperature distribution under these settings and the corresponding thermal damage (irreversible damage) of the inflammation are depicted at the inflammation center point on the XZ plane (Fig. 5) and the YZ plane (Fig. 6).

In both Figs. 5 and 6, the black line represents where the value of the Arrhenius damage integral (${\Omega }$) equals 1, indicating that irreversible damage occurs where ${\Omega }$ exceeds 1. As observed in these figures, under the same laser power (Figs. 5 and 6 (a, b,c), (d, e,f), (g, h,i)), an increase in the laser irradiation time resulted in an increase in the temperature of the inflammation at the same location, and an expansion of the area where irreversible damage occurred.

Examining the temperature distribution within the inflammation, it was observed that the temperature spread in a slightly distorted shape owing to the influence of the laser irradiation angle. Temperature diffusion is a phenomenon that propagates in all directions. In the case of inflamed tissues, which differ from typical solids in having relatively lower light absorption coefficients, laser light penetrates from the surface to the interior of the inflamed tissue. Consequently, the temperature also appeared to spread in the direction of laser irradiation, as calculated accurately. Under the same laser irradiation time (Figs. 5 and 6 (a, d,g), (b, e,h), (c, f,i)), as the laser power increased, the temperature of the inflammation increased and the area suffering thermal damage expanded. This indicated that, as the laser irradiation time and power increased, the extent of irreversible damage in inflammation increased. This can be interpreted as an increase in the total heat applied by the laser with longer irradiation times, allowing more time for heat to spread within the inflammation, thereby increasing the area of inflammation that undergoes irreversible damage. Additionally, as the laser power increased, the amount of heat applied per unit area to the inflammation increased, leading to an increase in the irreversible damage of inflammation. However, while inducing the irreversible damage of the inflammation is essential for the treatment of peri-implantitis, from the perspective of normal tissues, irreversible damage leads to tissue damage, and thus, an excessive temperature increase should be avoided.

**Fig. 7:**
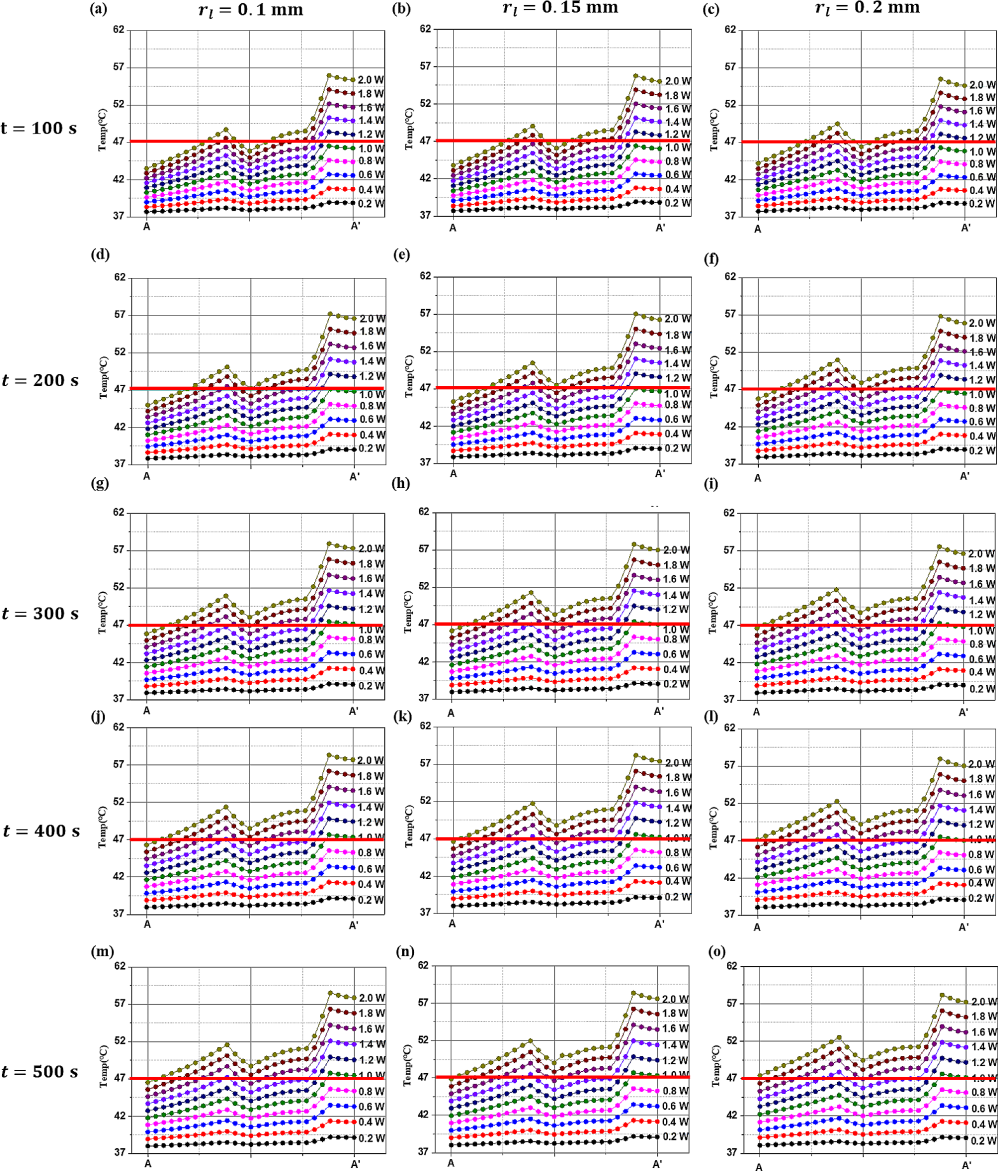
Temperature distribution of line AA’ with various radii and irradiation times of the laser

The implant surface temperature is directly related to the inflammation’s temperature. While laser energy absorption is localized within the inflammation, thermal conduction from the inflammation to implant can raises the implant surface temperature. The heat energy absorbed from the inflammation is transferred to the implant surface. If the temperature within the inflammation rises excessively due to overly intense laser power or prolonged irradiation times, it may result in the implant surface temperature exceeding the critical threshold of 47 ℃. This scenario poses a risk of thermal damage to the alveolar bone. Consequently, it is necessary to examine the temperature on the implant surface, which is the hottest part of the implant (AA’ line). As shown in Fig. 7, under all the laser radii and irradiation time conditions, the temperature at point AA’, where the implant physically contacts the inflammation and is the hottest, was examined. Below 1.0 W, even after *t* = 300 s, the implant surface temperature generally did not reach 47 ℃, except in some cases. However, above 1.0 W, the temperature of a significant portion of the implant surface exceeded 47 ℃; thus, caution is warranted during treatment.

### Laser radius and power behavior analysis in inflammation

In Sect. 3.2, we analyzed the degree of irreversible damage and the temperature distribution of the inflammation according to the laser irradiation time and intensity in various planar directions (XZ direction, YZ direction). In Sect. 3.3, we fixed the laser intensity at 0.8 W and set the laser irradiation radius to 0.1 mm, 0.15 mm, and 0.2 mm, and the irradiation time to 100 s, 300 s, and 500 s. Subsequently, we plotted the temperature distribution and the corresponding results under these conditions.

**Fig. 8:**
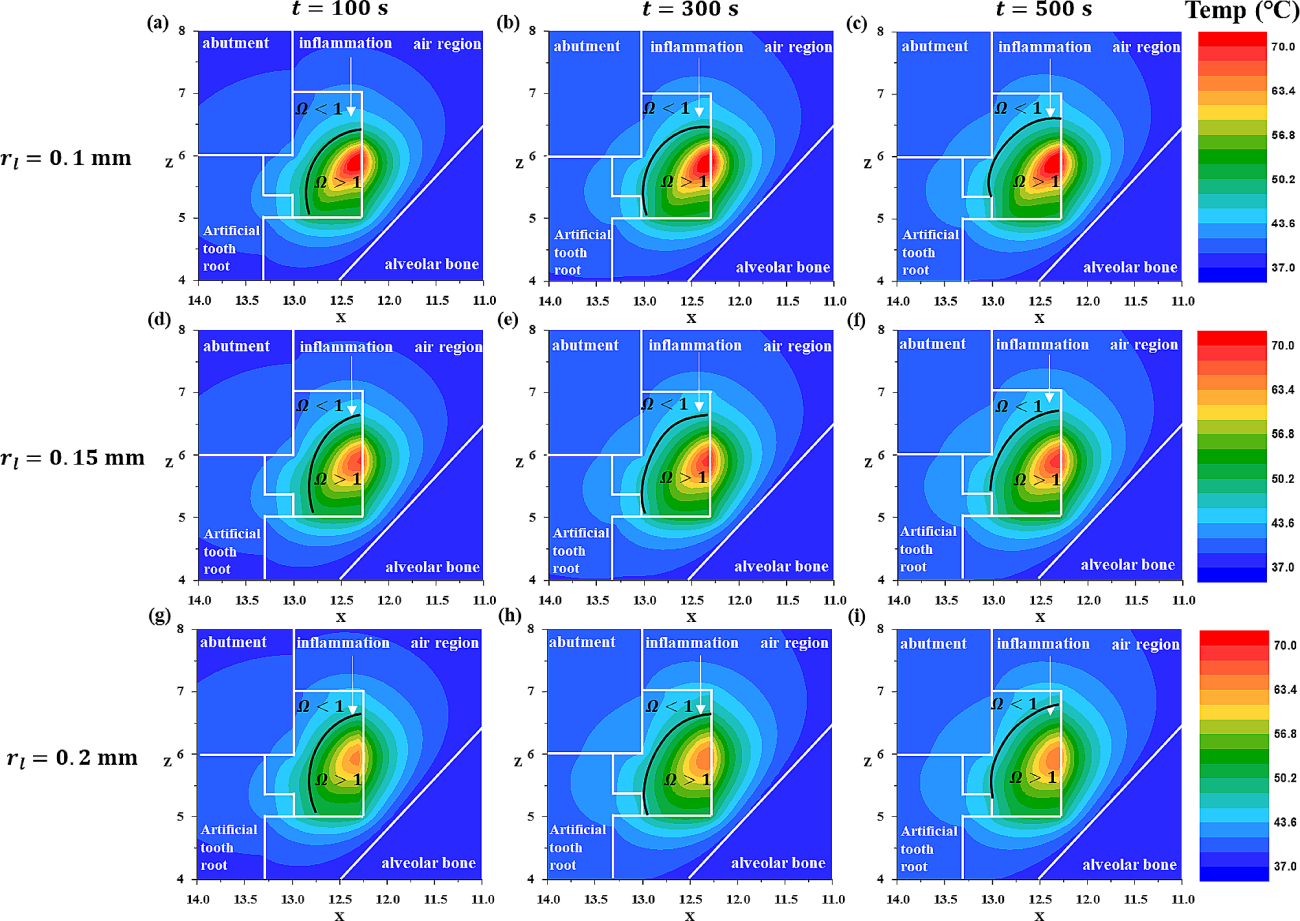
Temperature and ${\Omega }$ distribution with laser irradiation at various laser irradiation radii and laser irradiation times (XZ plane direction) for a laser power of 0.8 W

**Fig. 9:**
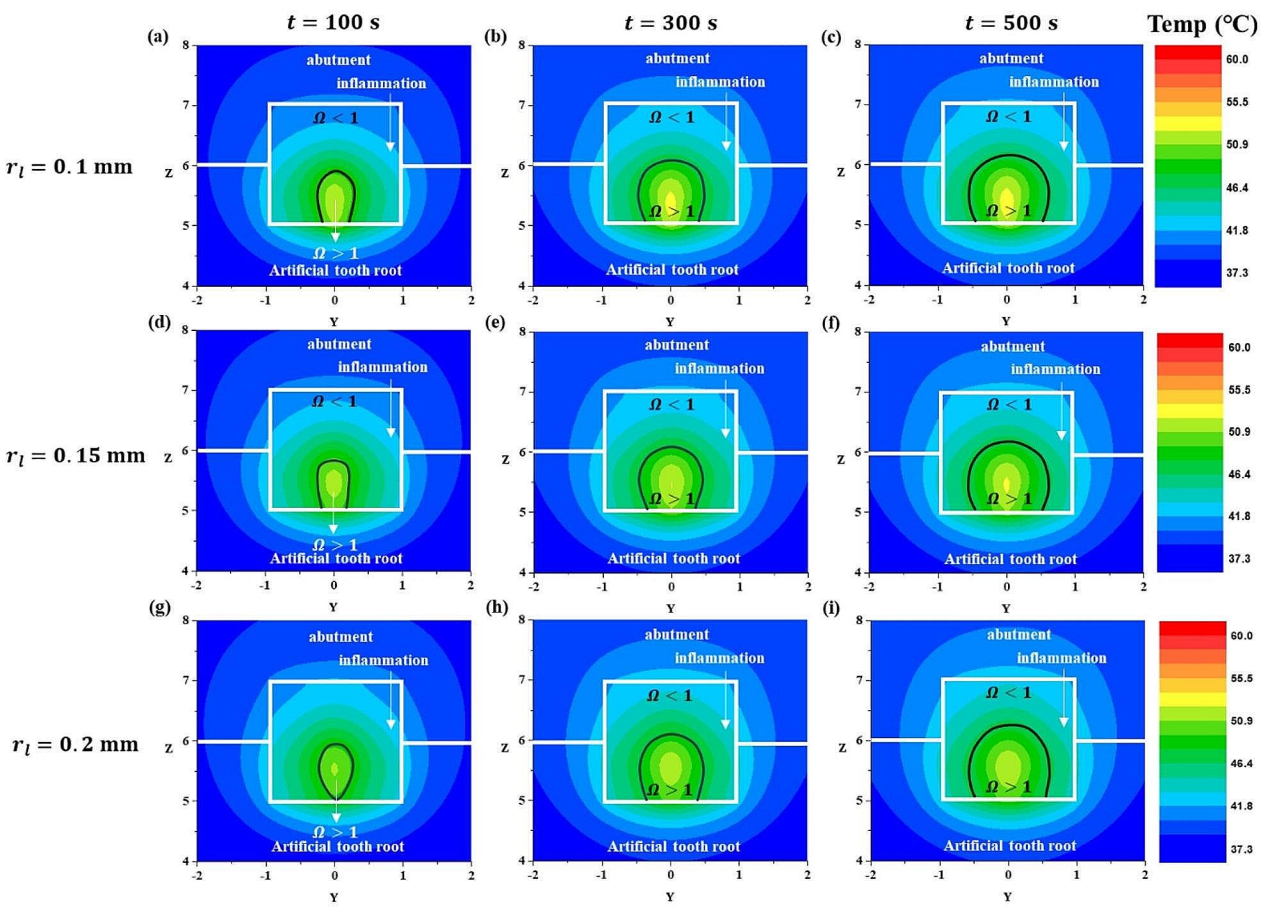
Temperature and ${\Omega }$ distribution with laser irradiation at various laser irradiation radii and laser irradiation times (YZ plane direction) for a laser power of 0.8 W

Figure 8 shows the temperature distribution results and the corresponding thermal damage to the inflammation when the shape in Fig. 4a is cut along the XZ plane at the inflammation center point. Figure 9 shows the temperature distribution results and the thermal damage to the inflammation when the shape in Fig. 4a is cut along the YZ plane at the inflammation center point. As mentioned in Sect. 3.2, the black line represents where the Arrhenius damage integral value (${\Omega }$) is 1, and if ${\Omega }$ exceeds 1, it indicates irreversible damage. Observing Figs. 8 and 9, when the laser intensity was fixed, a smaller laser radius increased the intensity per unit area, concentrating the temperature increase at the center of the inflammation. Conversely, as the laser radius increased, the area covered by the laser also increased, showing a similar trend in the extent of irreversible damage of inflammation across different laser radii (Figs. 8 and 9 (a, b,c), (d, e,f), (g, h,i)).

However, as will be discussed in Sect. 3.4, with laser powers above 0.8 W, the Arrhenius thermal damage ratio (${\phi }_{Arrh}$) varied depending on the radius as the laser power increased. Additionally, at the same laser power, an increase in the irradiation time led to an increase in the area of irreversible damage of inflammation for all laser radii, as calculated. As shown in Fig. 7, the temperature difference at AA’ for each radius is negligible, and at 0.8 W, there is no impact on the alveolar bone. However, as previously described, although the ${\phi }_{Arrh}$ value increases with increasing laser intensity, caution is required during treatment because of its potential impact on the alveolar bone.

### Arrhenius thermal damage ratio (${\phi}_{Arrh}$) in inflammation

In Sect. 3.2 and 3.3, we examined the extent of reversible damage and temperature distribution in the XZ and YZ planes inside the inflammation. In Sect. 3.4, we assessed the extent of irreversible damage throughout the entire volume of inflammation. As mentioned in Sect. 2.2, to evaluate the extent of damage across the entire inflammation, we used the Arrhenius thermal damage ratio (${\phi }_{Arrh}$), which, as Eq. (8) shows, represents the ratio of the volume in which the value of the Arrhenius damage integral exceeds 1 to the total volume of the inflammation.

**Fig. 10:**
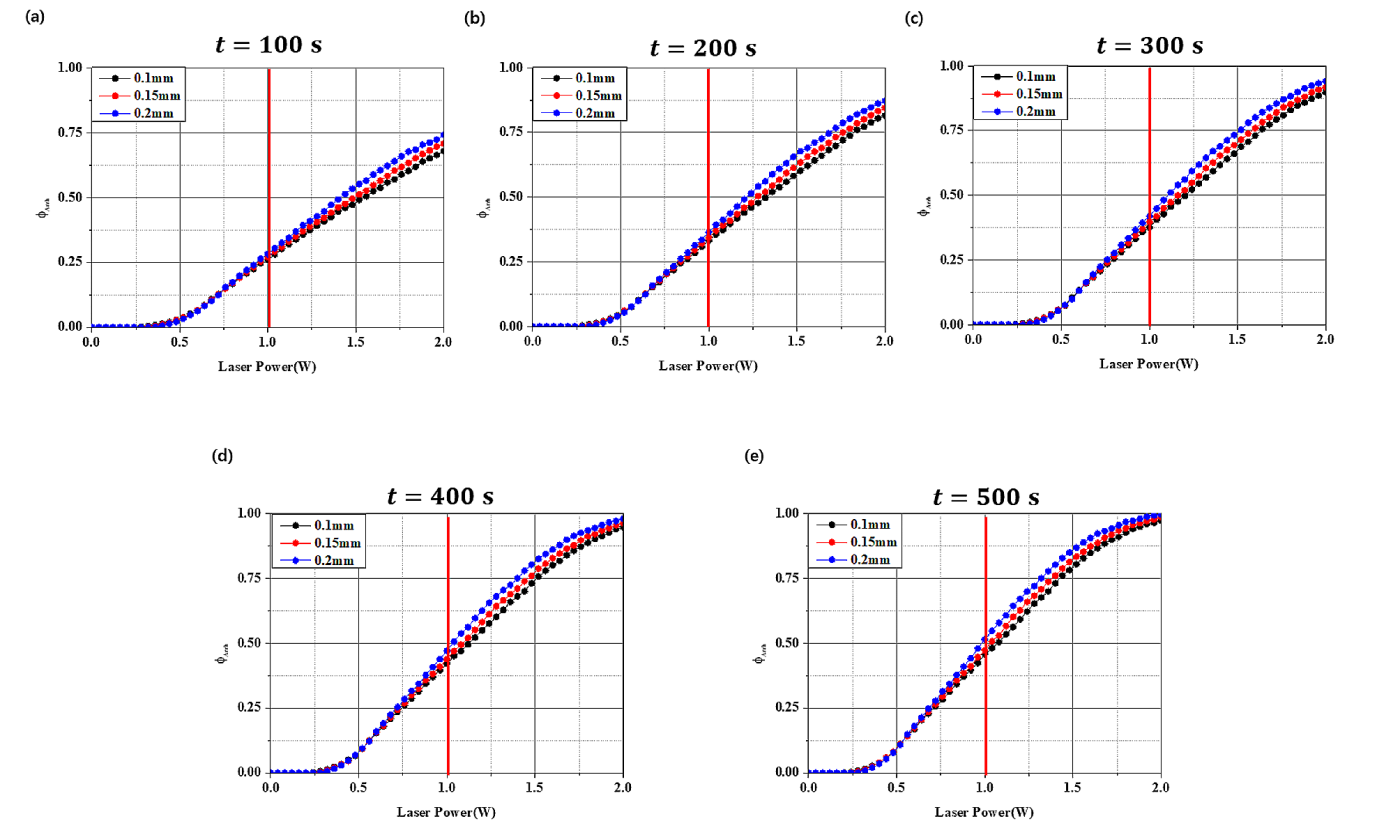
Arrhenius thermal damage ratio for various laser irradiation times

(a) laser irradiation time: 100 s, (b) laser irradiation time: 200 s, (c) laser irradiation time: 300 s, (d) laser irradiation time: 400 s, and (e) laser irradiation time: 500 s.

Figure 10 shows the values of ${\phi }_{Arrh}$ for different laser radii as the laser irradiation time increases, with the red line representing the point at 1.0 W. For all the laser radii, the ${\phi }_{Arrh}$ value increased as both the irradiation time and laser intensity increased. Notably, as the laser intensity increased, the variation in ${\phi }_{Arrh}$ values depending on the laser radius also became larger. In addition, at t = 100 s, the ${\phi }_{Arrh}$ value was approximately 0.75 for the maximum laser intensity (2.0 W) at a laser radius of 0.2 mm. This indicated that approximately 75% of the inflammation was eradicated. At t = 500 s, with the maximum laser intensity (2.0 W), the ${\phi }_{Arrh}$ value was close to 1, indicating that almost all the inflammation was eradicated. It is necessary to examine the ${\phi }_{Arrh}$ values at laser intensities below 1.0 W, where the temperature at the surface (AA’ line) where the inflammation and the implant meet approaches 47 ℃ (as shown in Fig. 7). Thus, the ${\phi }_{Arrh}$ value obtained at a laser irradiation time of 100 s was approximately 0.26, and it reached approximately 0.5 when the irradiation time was extended to 500 s.      

**Fig. 11:**
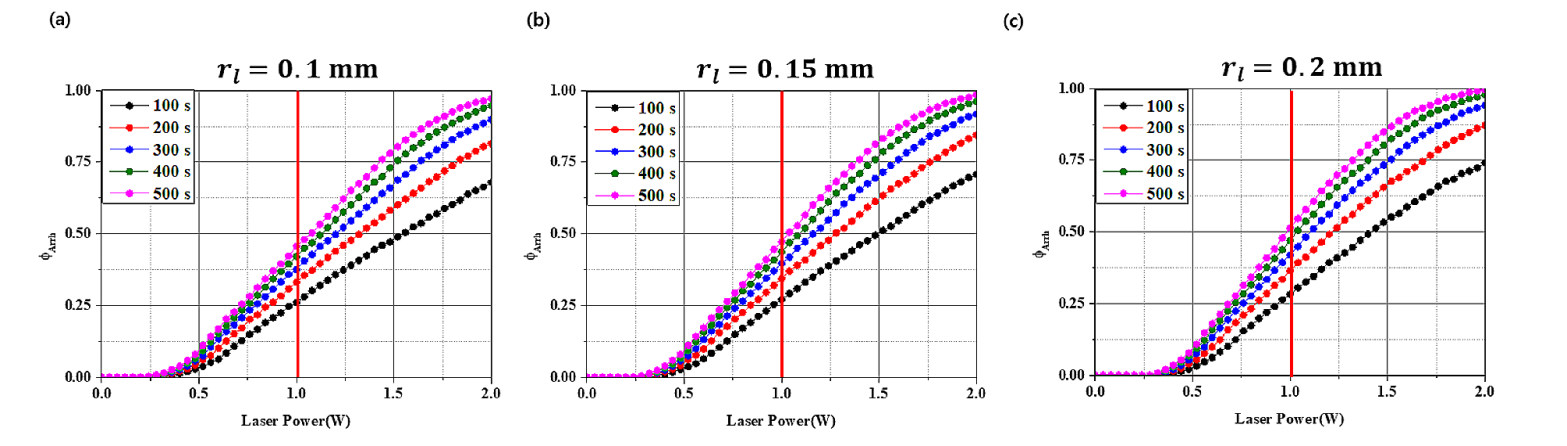
Arrhenius thermal damage ratio for various laser irradiation radii (a) laser irradiation radius: 0.1 mm, (b) laser irradiation radius: 0.15 mm, and (c) laser irradiation radius: 0.2 mm

In Fig. 11, the effect of the laser radius on ${\phi }_{Arrh}$ is depicted. With increasing laser radius, the ${\phi }_{Arrh}$ value increased for the same laser irradiation duration. Furthermore, the variation in ${\phi }_{Arrh}$ values for each irradiation time became more pronounced at higher laser intensities, and this amplification was notably greater than that caused by changes in the laser radius.

The values of ${\phi }_{Arrh}$ are similar to the results shown in Fig. 10, where conditions close to ${\phi }_{Arrh}$ being 1 were obtained at the laser intensity of 2.0 W, the irradiation time of 500 s, and the laser radii of 0.15 mm and 0.2 mm. In addition, at a laser intensity less than 1.0 W, a maximum ${\phi }_{Arrh}$of approximately 0.5 was achieved. Note that, as shown in Fig. 7, the AA’ line (the line where the inflammation contacts the implant) is more than 2 mm away from the alveolar bone. Therefore, the temperature rise along the AA’ line undergoes a conduction process from the artificial tooth root to the alveolar bone, and the temperature at the contact area between the alveolar bone and the artificial tooth root is lower. Further analysis could lead to the discovery of laser intensities higher than that (1.0 W) set in Figs. 10 and 11 to avoid thermal damage to the alveolar bone, potentially achieving higher${\phi}_{Arrh}$ values without causing thermal damage to the alveolar bone.      

## Discussion

This numerical study identifies optimal laser irradiation conditions that effectively treat inflammation around implants while minimizing damage to the alveolar bone, through a numerical analysis of the effects of laser intensity, radius, and irradiation time on temperature distribution. By understanding the impact of these variables on temperature distribution, the numerical study lays a foundation for managing the risk of thermal damage, crucial in implant therapy.

The temperature distribution in the biological tissues and implants was determined using the Pennes bioheat equation, and the heat from the laser was modeled by modifying the existing laser heat formula to consider tilted laser irradiation. The wavelength of the laser was 630 nm, and the angle of the laser was fixed at 60°. Additionally, by utilizing the Arrhenius damage integral, which was calculated from the temperature distribution to assess the PTT effect quantitatively, the analysis differentiated between areas that underwent irreversible damage and those that did not. Finally, using the Arrhenius thermal damage ratio (${\phi }_{Arrh}$), the extent of irreversible damage throughout the entire inflammation was calculated under all the analysis conditions.

From the analysis results, at a laser intensity of 0.8 W or less, the variation in ${\phi}_{Arrh}$values based on the laser radius was minimal. However, at laser intensities above 0.8 W, a greater deviation in ${\phi}_{Arrh}$values were observed as the laser radius increased. Additionally, the increase in ${\phi}_{Arrh}$values was quantitatively assessed as the laser irradiation time increased. The temperature distribution on the surface (AA’ line) where the inflammation contacts the implant was also analyzed to consider the thermal damage to the alveolar bone. Based on the analysis of the implant surface (AA’ line) temperature, it was confirmed that the thermal conduction process from the artificial tooth root causes no impact on the alveolar bone, which is situated more than 2 mm away. Consequently, conditions that could completely eradicate the inflammation without affecting the alveolar bone were identified. Moreover, the critical observation that the AA’ line’s temperature (the interface between the implant and inflammation) does not reach the temperature threshold when using specific laser conditions emphasizes the precision with which laser therapy can be applied. Specifically, our findings at laser intensities below 1.0 W, which prevented the temperature from exceeding the critical threshold of 47 ℃, highlight the potential for optimizing laser conditions to maximize therapeutic effects while safeguarding alveolar bone.

By exploring the potential benefits of photothermal therapy as a non-surgical treatment method for peri-implantitis, our findings offer promising insights into the application of photothermal therapy in clinical settings. By identifying laser irradiation conditions that effectively treats inflammation without thermal damage to the alveolar bone, the results of this numerical study suggest that more rigorous treatments can be performed in practical peri-implantitis laser therapy. Specifically, by establishing laser irradiation guidelines that prevent alveolar bone damage while effectively treating inflammation through precise thermal management, the numerical study represents significant progress in the field of dental implants. One limitation of this numerical study is that the numerical analysis model may not fully implement the complexity of actual biological conditions. The parameters assumed in the modeling process might not consider all the variables present in practical clinical setting. Future studies could be of significant value, including investigations of thermal conduction through the artificial tooth root, the distance between the alveolar bone and the laser irradiation site, changes in laser irradiation angle, various shapes of inflammation, and expanding the analysis of irreversible eradication conditions to not only inflammation, but also surrounding tissues, such as bones and gingiva.

## Conclusions

This research establishes a fundamental comprehension of the optimization of photothermal therapy for effective treatment of peri-implantitis, with a concurrent focus on preserving the alveolar bone integrity against thermal damage. As the results, the Arrhenius thermal damage ratio (${\phi}_{Arrh}$), the extent of irreversible damage throughout the entire inflammation was calculated under various the laser intensity, laser radius and laser irradiation times. Our findings highlight the promise of non-surgical photothermal therapy in dental implantology, which provides a precise, regulated way for treating peri-implant inflammation. It is expected that the results and numerical analysis model presented in this numerical study will aid in establishing a method for the thermal adjunctive treatment of peri-implantitis.

## Data Availability

All data generated or analyzed in this study are included in this published article.

## Nomenclature

*A*: frequency factor (1/s)
*c*_*p*_: specific heat (J/kgK)
$$ {E}_{a}$$: activation energy (J/mol)
*k*: thermal conductivity (W/mK)
*P*_*l*_: intensity of laser (W)
$$ q$$: volumetric heat source (W/m^3^)
$$ {r}_{l}$$: laser radius (mm)
$$ {R}_{t}$$: reflectivity
$$ R$$: ideal gas constant (J/molK)
*T*: temperature (K)
*t*: laser irradiation time (s)

## Greek symbols

$$ \theta $$: irradiation angle
*µ’*: reduced optical coefficient (1/cm)
$$ \rho $$: density (kg/m^3^)
${\phi }_{Arrh}$: Arrhenius thermal damage ratio
*ω*_*b*_: blood perfusion rate (1/s)
${\Omega }$: Arrhenius damage integral value
*µ*: optical coefficient (1/cm)

## Subscripts

*a*: absorption
*b*: blood
*l*: laser
*m*: metabolic
*s*: scattering
*tot*: total
*x, y, z*: notation of direction
